# Delayed Yet Successful Mechanical Thrombectomy for Phlegmasia Cerulea Dolens in a Limb with Severe Arterial Disease and May-Thurner Syndrome

**DOI:** 10.1155/2020/8866030

**Published:** 2020-11-01

**Authors:** Ahmad Said, Phillip Kraft, Luay Sayed

**Affiliations:** ^1^Department of Internal Medicine, Beaumont Health, Troy, MI, USA; ^2^Oakland University William Beaumont School of Medicine, Rochester, MI, USA; ^3^Department of Cardiovascular Medicine, Beaumont Health, Troy, MI, USA

## Abstract

Phlegmasia cerulea dolens (PCD) is a rare but life-threatening complication of acute deep venous thrombosis that lacks consensus regarding the approach to management. We present a case of PCD developing shortly after a spinal surgery and manifesting as acute swelling and discoloration in a leg with existing severe atherosclerotic arterial disease. The patient's critical limb ischemia was completely and rapidly reversed by percutaneous mechanical thrombectomy using the ClotTriever device despite a delay in treatment. An underlying iliac vein compression “May-Thurner” syndrome was discovered using intravascular ultrasound and treated with angioplasty. This case identifies mechanical thrombectomy using the ClotTriever system as a possible effective and safe treatment for PCD.

## 1. Introduction

Phlegmasia cerulea dolens (PCD) “painful blue edema” is an uncommon condition that results from acute massive deep venous thrombosis (DVT) of an extremity and is characterized by total venous blood flow obstruction leading to an increase in compartmental pressure that eventually results in arterial blood flow compromise, limb ischemia, circulatory collapse, and shock, especially if diagnosis and/or management is delayed. Reported limb amputation rates range from 12% to 50%, and mortality ranges from 25% to 40% [1], making PCD a medical emergency. Due to the rarity of PCD and the lack of randomized controlled trials, there is no gold standard regarding its management. Treatment options include anticoagulation, systemic thrombolytics, catheter-directed thrombolysis (CDT), pharmacomechanical CDT, surgical thrombectomy, and percutaneous thrombectomy. Although anticoagulation with intravenous (IV) heparin is usually the first line of treatment in the absence of contraindications, the subsequent treatment approach is controversial, and there are no guidelines to aid physicians in that process.

## 2. Case Presentation

**Figure 1:**
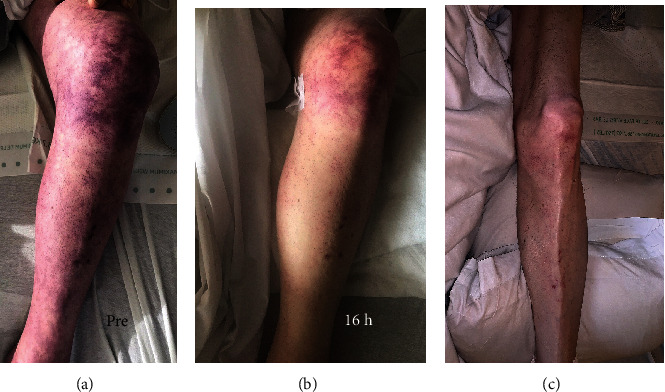
Left lower extremity: (a) at the time of diagnosis of phlegmasia cerulea dolens; (b) 16 hours after thrombectomy; (c) at one-month follow-up.

**Figure 2:**
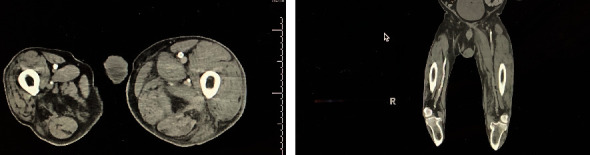
Computed tomography angiography showing the lack of arterial blood flow in the left superficial femoral and proximal popliteal arteries.

A 78-year-old man with a history of severe peripheral arterial disease (PAD) requiring left third toe amputation and percutaneous stenting of the left superficial femoral artery was admitted to the intensive care unit (ICU) due to an acute subarachnoid hemorrhage (SAH) which occurred after a fall. The patient was also found to have an acute fracture in the tenth thoracic vertebra, and he subsequently underwent spinal fusion of the eighth to twelfth thoracic vertebrae. Within three hours postoperation, the patient was still lethargic and did not complain of pain; however, his left lower extremity was noted to be swollen and cool to the touch from the thigh down with a mottled skin appearance (Figure 1(a)). Pedal pulses were completely absent on palpation, and no signal was detected on a portable Doppler device. The initial suspicion was for an arterial embolic event causing limb ischemia as the patient had a history of atrial fibrillation. As a result, a computed tomography arteriography (CTA) of the abdominal aorta with bilateral runoff was done and showed the previously placed stent extending from the left superficial femoral artery to the proximal popliteal artery with absent filling consistent with vascular occlusion (Figure 2). Due to significant leg swelling, a venous duplex ultrasound was done and revealed extensive acute deep vein thrombosis (DVT) ranging from the left common femoral vein down to the popliteal vein with completely absent venous blood flow. The clinical and radiographic findings were consistent with a diagnosis of phlegmasia cerulea dolens (PCD).

**Figure 3:**
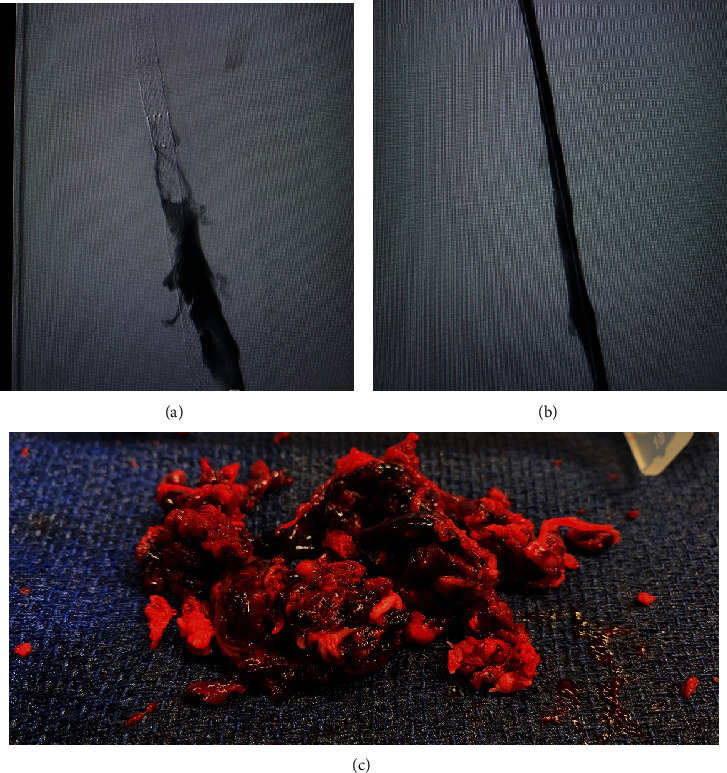
Venography of the lower extremity: (a) absence of blood flow due to thrombus; (b) restoration of blood flow after thrombectomy; (c) large amount of thrombus extracted using the ClotTriever system.

**Figure 4:**
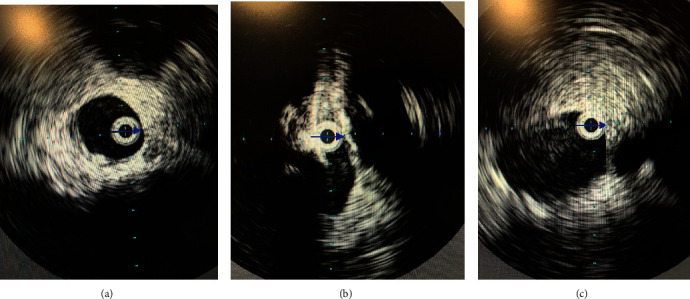
Intravascular ultrasound (IVUS) of the iliac vein: (a) precompression; (b) site of compression; (c) postcompression.

**Figure 5:**
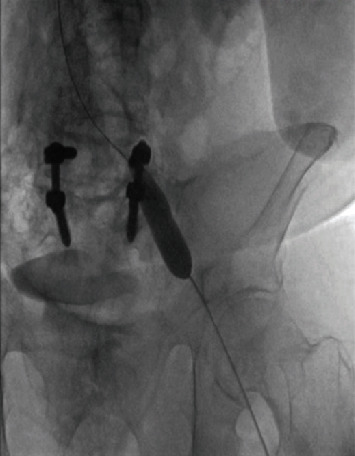
Fluoroscopy showing percutaneous transluminal angioplasty of the left iliac vein.

After clearance from the neurosurgeon, IV heparin was started approximately 7 hours after the development of symptoms. Due to the extensive DVT and the advanced PAD, the vascular surgery team deemed that the chances of limb salvage are very small and that any procedural intervention will pose a great risk to the patient's life. Thrombolysis was contraindicated due to the recent SAH and spinal surgery. After comprehensive multidisciplinary discussions including a second expert opinion which was sought from a peripheral interventional cardiologist, a delayed decision was reached to attempt a minimally invasive percutaneous limb-salvaging approach which occurred 20 hours after the diagnosis was established. In the catheterization lab, venography of the inferior vena cava (IVC) via left popliteal vein access revealed extensive and occlusive thrombosis of the iliac, femoral, and popliteal veins (Figure 3(a)). The IVC was patent. Doppler ultrasound confirmed the absence of blood flow in the popliteal artery. Under ultrasound guidance, the left popliteal vein was accessed, and the ClotTriever (Inari Medical) sheath was placed. The ClotTriever thrombectomy catheter was then introduced, and six device passes were made extending from the iliac vein back into the popliteal vein with extraction of a significant amount of thrombus (Figure 3(c)). An intravascular ultrasound detected compression strictures of the common iliac and femoral veins (Figure 4), and percutaneous transluminal angioplasty was performed (Figure 5). Immediate postintervention venography revealed restoration of the venous flow (Figure 3(b)), and ultrasound Doppler detected a strong popliteal arterial flow. Sixteen hours postintervention, the leg swelling and discoloration almost completely resolved (Figure 1(b)). At 1-month follow-up, the left leg had completely returned to baseline appearance without any evidence of edema or discoloration (Figure 1(c)). No amputation was needed. The patient reported in this article has provided consent to publish their case details and images.

## 3. Discussion

Phlegmasia cerulea dolens (PCD) is a venous disaster with high rates of morbidity and mortality, yet with no evidence-based guidelines for management. After extensive literature review, only a few case reports were identified, and there were no studies favoring one treatment approach over the other. Gociman et al. describe a case of PCD treated with a combination of surgical and CDT [2]. Two other published cases describe a different combination therapy approach with pharmacomechanical CDT in patients who were at high risk for bleeding from systemic thrombolytics [3, 4]. The patient in our case had an absolute contraindication to thrombolytics because of the recent SAH and spinal surgery. He also had severe PAD in the affected limb with total occlusion of the posterior tibial artery at baseline. Due to his medical history and high surgical risk, the surgical team recommended against limb salvage. However, upon further discussions and after an interventional cardiology consultation, an attempt at mechanical percutaneous thrombectomy was considered a safe option. The patient's PCD was successfully treated using the ClotTriever system. Venous and arterial blood flow was immediately restored following the procedure despite the delay in intervention. The ClotTriever system has been validated in a few studies, including a recent small retrospective study evaluating short-term outcomes following treatment of extensive DVT's using the ClotTriever system. All patients had complete evacuation of the clot and resolution of symptoms, including one who had PCD [5]. The literature also described a few more cases where the ClotTriever system was successfully used for DVT. In one case study, it was used as an adjunct to CDT [6], and in two other cases, it was used as the sole treatment, one for a patient with extensive DVT [7] and another for a patient with PCD [8].

A compression of the common iliac vein, also known as May-Thurner syndrome, has been uncovered as a disposing factor for PCD in multiple reported cases [9, 10]. Stenting of the iliac vein is commonly performed in this scenario as an attempt to prevent future recurrences of DVT. Although our patient was also found to have iliac vein compression on intravascular ultrasound, stenting of the iliac vein was postponed as treatment with antiplatelets in addition to anticoagulation would have resulted in a significant risk of bleeding complications due to the patient's recent SAH and spinal surgery. Angioplasty was used instead as a temporary measure. Our patient's course was favorable following the procedure as the swelling resolved within a few days, and no amputation was required. During an office visit one month after thrombectomy, his lower extremity seemed to have returned to baseline in appearance and function.

## 4. Conclusion

This is a case of phlegmasia cerulea dolens that developed shortly following a spinal surgery in a limb with underlying arterial occlusive disease and May-Thurner syndrome. Due to contraindications to thrombolytics, a single approach via the ClotTriever thrombectomy device was successful in saving the limb despite the delay in treatment.

## Data Availability

All data are available upon request.

## Supplementary Materials

Supplementary Materials (Video): intravascular ultrasound of the iliac vein revealing extrinsic compression of the iliac vein by the iliac artery “May-Thurner syndrome.”
